# Improving the prediction of the trabecular bone microarchitectural parameters using dental cone-beam computed tomography

**DOI:** 10.1186/s12880-019-0313-9

**Published:** 2019-01-23

**Authors:** Rong-Ting He, Ming-Gene Tu, Heng-Li Huang, Ming-Tzu Tsai, Jay Wu, Jui-Ting Hsu

**Affiliations:** 1 School of Dentistry, College of Dentistry, China Medical University, Taichung, 404 Taiwan, Republic of China; 2 Department of Dentistry, China Medical University and Hospital, 91 Hsueh-Shih Road, Taichung, 404 Taiwan, Republic of China; 3 Department of Bioinformatics and Medical Engineering, Asia University, Taichung, 413 Taiwan, Republic of China; 4 Department of Biomedical Engineering, Hungkuang University, Taichung, 433 Taiwan, Republic of China; 5 Department of Biomedical Imaging and Radiological Sciences, National Yang-Ming University, Taipei, 112 Taiwan, Republic of China

**Keywords:** Dental cone-beam computed tomography, Micro-computed tomography, Trabecular bone microarchitectural parameters, Image preprocessing approach

## Abstract

**Background:**

In this study, we explored how various preprocessing approaches can be employed to enhance the capability of dental CBCT to accurately estimate trabecular bone microarchitectural parameters.

**Methods:**

In total, 30 bovine vertebrae cancellous bone specimens were used for in study. Voxel resolution 18-μm micro-computed tomography (micro-CT) and 100-μm dental CBCT were used to scan each specimen. Micro-CT images were used to calculate trabecular bone microarchitectural parameters; the results were set as the gold standard. Subsequently, before the dental CBCT images were converted into binary images to calculate trabecular bone microarchitectural parameters, three preprocessing approaches were used to process the dental CBCT images. For Group 1, no preprocessing approach was applied. For Group 2, images were sharpened and despeckable noises were removed. For Group 3, the function of local thresholding was added to Group 2 to form Group 3. For Group 4, the air pixels was removed from Group 3 to form Group 4. Subsequently, all images were imported into a software package to estimate trabecular bone microarchitectural parameters (bone volume fraction (BV/TV), trabecular thickness (TbTh), trabecular number (TbN), and trabecular separation (TbSp)). Finally, a paired *t*-test and a Pearson correlation test were performed to compare the capability of micro-CT with the capability of dental CBCT for estimating trabecular bone microarchitectural parameters.

**Results:**

Regardless of whether dental CBCT images underwent image preprocessing (Groups 1 to 4), the four trabecular bone microarchitectural parameters measured using dental CBCT images were significantly different from those measured using micro-CT images. However, after three image preprocessing approaches were applied to the dental CBCT images (Group 4), the BV/TV obtained using dental CBCT was highly positively correlated with that obtained using micro-CT (*r* = 0.87, *p* < 0.001); the correlation coefficient was greater than that of Group 1 (*r* = −0.15, *p* = 0.412), Group 2 (*r* = 0.16, *p* = 0.386), and Group 3 (*r* = 0.47, *p* = 0.006). After dental CBCT images underwent image preprocessing, the efficacy of using dental CBCT for estimating TbN and TbSp was enhanced.

**Conclusions:**

Image preprocessing approaches can be used to enhance the efficacy of using dental CBCT for predicting trabecular bone microarchitectural parameters.

## Background

Bone comprises external cortical bone and internal cancellous bone. Trabecular bone is the central part of cancellous bone. Trabecular bones of various thickness and porosity levels or in various directions can form cancellous bones at various structural stiffness levels [1]. Trabecular bone microarchitecture is a crucial indicator for assessing trabecular bone mechanical property [1–4]. Previously, bone histomorphometry was typically employed to measure trabecular bone microarchitecture. Although bone histomorphometry can be used to accurately measure trabecular bone microarchitecture, it can only obtain two-dimensional sections and the testing method is typically considered invasive [5, 6]. Over the past two decades, micro-computed tomography (micro-CT) has been considered a gold standard for assessing trabecular bone microarchitecture [5, 7]. In addition, numerous trabecular bone microarchitectural parameters must be considered (e.g., bone volume fraction (BV/TV), trabecular bone thickness (TbTh), trabecular bone separation (TbSp), trabecular bone number (TbN), bone surface, bone surface density, connectivity density, structure model index, degree of anisotropy, and mean intercept length.) [7, 8]. In 2009, Bouxsein et al. [7] identified BV/TV, TbTh, TbN, and TbSp as the basic parameters for analyzing trabecular bone microarchitecture. Although micro-CT is the gold standard for assessing the trabecular bone microarchitectural parameters, the clinical application of micro-CT is limited because of its narrow scan field and high radiation dose.

Dental cone-beam computed tomography (dental CBCT) has increasingly been applied in dental clinics for oral and maxillofacial surgery, endodontics, implantology, orthodontics, temporomandibular joint dysfunction, periodontics, restorative work, and forensic dentistry [9–13]. Compared with conventional CT, dental CBCT features the following advantages: lower radiation dose, shorter acquisition times, higher resolution, and affordability [9, 10, 13]. Dental CBCT is frequently employed to assess the bone quality and quantity of the jawbone prior to dental implant surgery. Some researchers have indicated that the bone density grayscale value for cancellous bone obtained through dental CBCT were lower than the bone density in Hounsfield units (HU) obtained through CT. However, because dental CBCT hardware has improved, researchers have increasingly adopted dental CBCT to assess the bone density of the jawbone prior to dental implant surgery [14–16].

In recent years, dental CBCT resolution has improved. Resolution of some dental CBCT can reach 80 μm in the scanning mode for a narrow field of scan [17]. However, their resolution remains unsuitable for accurately measuring trabecular bone microarchitectural parameters. According to previous studies, the TbTh of trabecular bone obtained through dental CBCT was greater than that obtained through micro-CT primarily because dental CBCT did not possess satisfactory resolution. When measuring 200–400-μm thick trabecular bone [18], the TbTh obtained using dental CBCT was overestimated because of a partial volume effect; accordingly, other trabecular bone microarchitectural parameters were estimated incorrectly. In addition, during dental CBCT scans, air, body fluids, or soft tissue inside cancellous bone can cause image noise and influence the measurement of trabecular bone microarchitecture. According to previous studies in which micro-CT was applied to measure trabecular bone microarchitectural parameters, various binary-image methods with various threshold values for segmenting the trabecular bone images influenced the measurement results [19–21]. Numerous studies have explored the effect of various dental CBCT scan protocols (e.g., object location, scan resolution, exposure time, and electric current) on measurement outcomes. However, few studies have investigated the effects of using various binary image processing methods for dental CBCT before trabecular bone microarchitectural parameter measurements.

Numerous studies have indicated that trabecular bone parameters obtained using dental CBCT and micro-CT would be significantly different [2, 14, 22–24]; in addition, various studies have presented various conclusions concerning the correlations between trabecular bone microarchitectural parameters estimated from dental CBCT and those estimated from micro-CT [2, 14, 22–24]. Some studies have determined that estimates of trabecular bone microarchitectural parameters obtained through dental CBCT were highly correlated with those obtained through micro-CT. However, some other studies have identified no substantial correlation between the estimates of trabecular bone microarchitectural parameters obtained through dental CBCT and those obtained through micro-CT. In the present study, we used bovine vertebrae cancellous bone specimens as samples. We first measured the trabecular bone microarchitectural parameters from the image obtained using micro-CT. Subsequently, we used several image preprocessing approaches to explore the capacity of dental CBCT to measure trabecular bone microarchitectural parameters.

## Methods

### Specimen preparation

In this study, we used 30 bovine vertebrae cancellous bone specimens as samples (the size of each sample was 20 × 20 × 20 mm^3^). All bovine vertebrae cancellous bone specimens were obtained from a local meat market. Each sample was wrapped with wet gauze and medical tape and subsequently placed in a zipper bag and frozen at − 20 °C in a constant-temperature refrigerator. Before imaging, all samples were placed in a room-temperature environment and defrosted for 8 h. To align the regions of interest (ROI) in micro-CT and dental CBCT images, a dental composite resin ball was placed on the top surface of each bone specimen to serve as an anchor point. In addition, when dental CBCT was employed to image each bone specimen, a hydroxyapatite phantom (QRM GmbH, Möhrendorf, Germany) was used as a reference for adopting an image preprocessing approach to remove air pixels.

### Micro-CT and dental CBCT scanning

**Fig. 1:**
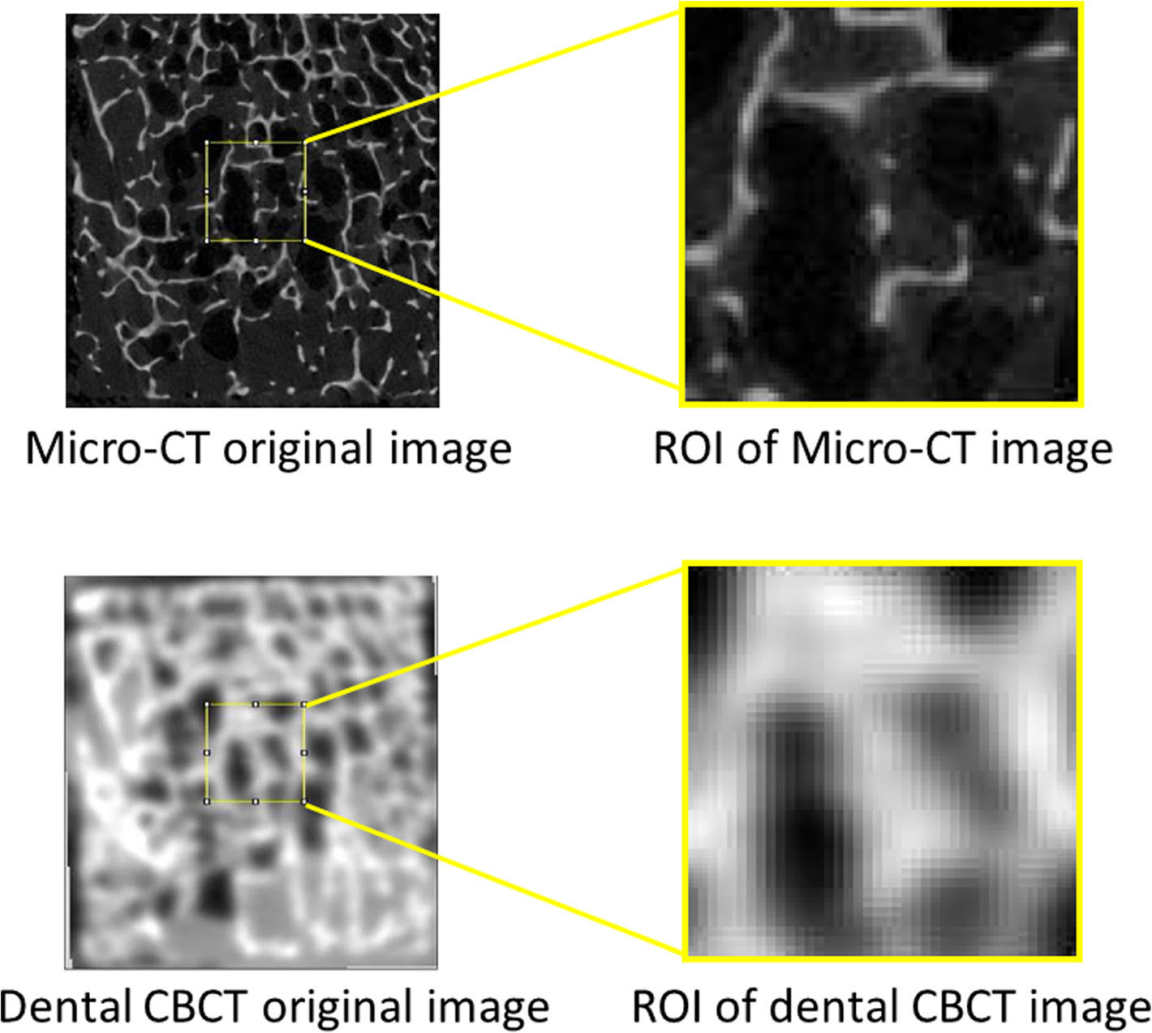
Original and ROI images of the two scanning methods: (top) micro-CT image; (bottom) dental CBCT image

Micro-CT images were obtained using a Skyscan 1076 micro-CT (SkyScan, Aartselaar, Belgium). The scanning parameters were set to 80 kV, 200 μA, and a voxel resolution of 18 μm. The exposure time was 450 milliseconds. The micro-CT scanning images were saved in the TIF file format. After scanning, the images were imported into NRecon (SkyScan, Aartselaar, Belgium) for image reconstruction. Dental CBCT images were scanned using AZ 3000 (Asahi Roentgen, Japan). The scanning parameters were set to 85 kV, 5 mA, and a voxel resolution of 100 μm. The exposure time was 17 s. The dental CBCT scanning images were saved as Digital Imaging and Communications in Medicine file format. Subsequently, dental CBCT and micro-CT images were imported into an image software package called ImageJ 1.46r (Rasband, W.S., ImageJ, U.S. National Institutes of Health, Bethesda, MD, USA). The position of the dental composite resin ball was used for alignment (Fig. 1). The alignment method was used to identify the surfaces of bone specimens in the dental CBCT and micro-CT images on which the composite resin balls were placed and then identify the central point of the surface (Fig. 1). Subsequently, a 4 × 4 × 10 mm^3^ cuboid was segmented from the central point inward to the bone specimen to serve as the ROI, the size of which approximated that of the dental implant.

### Image preprocessing of micro-CT and dental CBCT

**Fig. 2:**
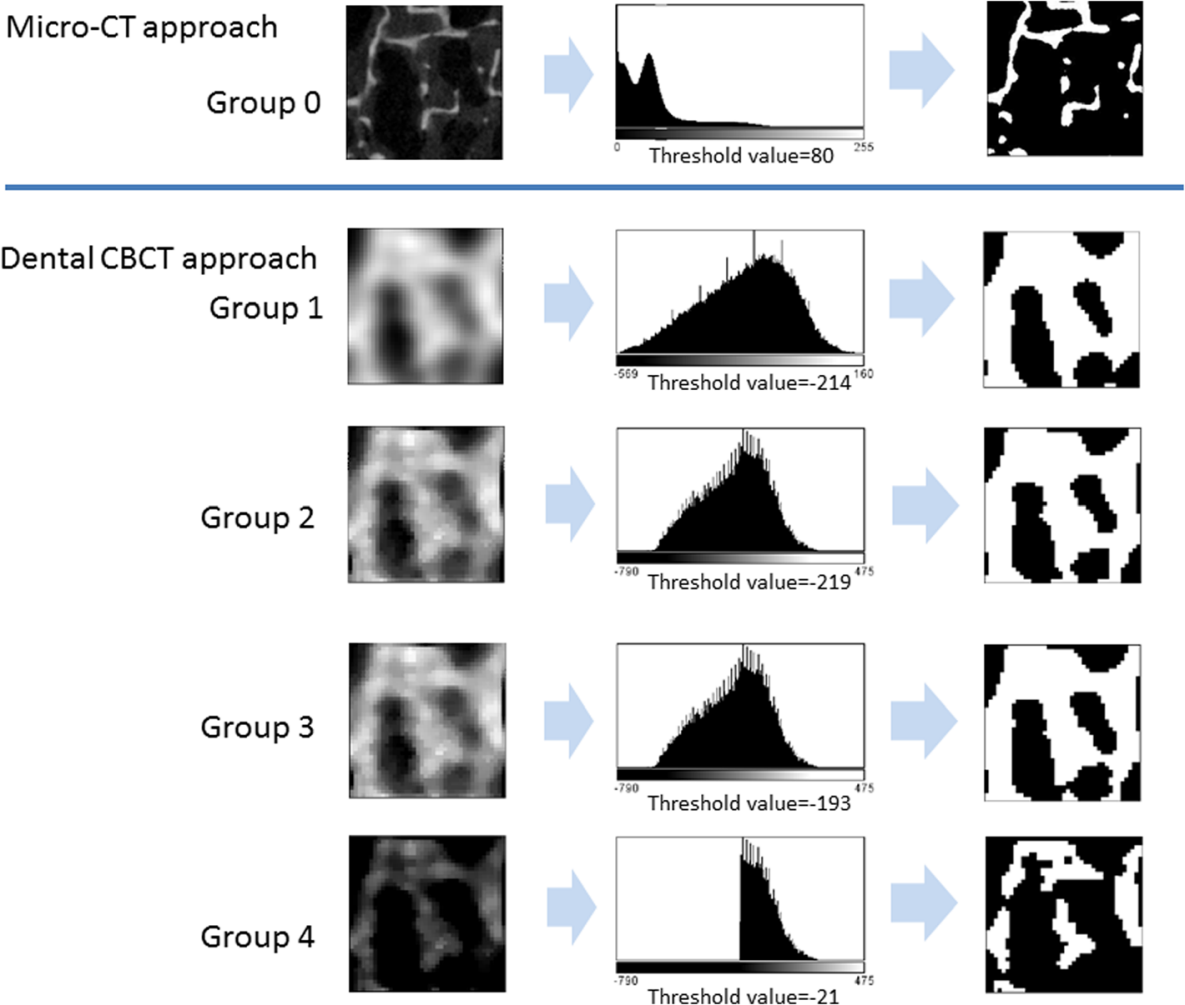
Preprocessing of micro-CT (group 0) and dental CBCT (Group 1 to 4) images before binary image conversion for calculating trabecular bone microarchitectural parameters

In recent years, micro-CT has been considered the gold standard for assessing trabecular bone microarchitecture. Therefore, in this study, the micro-CT images did not undergo image preprocessing and were directly converted into binary images to measure trabecular bone microarchitectural parameters. The measurement results were called Group 0, which served as the gold standard for assessing dental CBCT capability (Fig. 2). This study explored three dental CBCT image preprocessing approaches used in four groups, namely control Group 1 and experimental Groups 2 to 4. Three preprocessing steps were followed as detailed hereafter. Images from Groups 1 to 4 were converted into binary images with ImageJ software (Fig. 2).

Group 1: dental CBCT images did not undergo any image preprocessing.

Group 2: Group 1 images underwent sharpening filter and despeckable filter. The ImageJ software default method was used to sharpen the images by increasing the contrast and accentuating the details in the image. The despeckable filter replaced each pixel with the median value in its 3 × 3 neighborhood.

Group 3: Images were converted into binary images with a local threshold, rather than the Group 2 and Group 1 using global threshold. For Group 2 and Group 1, the global binary threshold was calculated using an isodata algorithm from the whole image data. However, this threshold would overestimate thin trabecula in the low-density image slice and underestimate thick trabecula in the high-density slice. The local threshold method could calculate the threshold by each slice data and achieve much more proper trabecula contour.

Group 4: The suppositional air images in Group 3 were removed. We initially used HA phantom images to calculate the correlation between specimen bone mineral density and image grayscale values. Subsequently, the images in which grayscale values corresponded to zero bone mineral density were supposed to be air or unnecessary images that should be removed.

### Trabecular bone microarchitectural parameters measured using micro-CT and dental CBCT

All micro-CT and dental CBCT images were imported into CTAn (SkyScan, Aartselaar, Belgium) to measure the following four trabecular bone microarchitectural parameters: BV/TV, ratio of the segmented trabecular bone volume to the total trabecular volume, (unit = %); TbTh, mean trabecular thickness, (unit = millimeter); TbN, average number of trabecula per millimeter, (unit = trabeculae per millimeter); and TbSp, mean distance between trabecula, (unit = millimeter) of the ROI.

### Statistical analysis

The mean and standard deviation were calculated for all measurements. Paired *t*-tests were used to compare the differences between the dental CBCT and micro-CT measurements, and the significance level was set at 0.05. Pearson correlation was conducted to calculate the correlation coefficients (*r* values) between the dental CBCT and micro-CT measurements. All statistical analyses were performed using SPSS v. 19 (IBM Corporation, Armonk, NY, USA).

## Results

### Trabecular bone microarchitectural parameters determined using micro-CT and dental CBCT

Table 1 presents the estimates of the four trabecular bone microarchitectural parameters obtained through micro-CT and dental CBCT. Micro-CT analysis indicated that the BV/TV value was 23.85 ± 7.83% (Group 0). By applying image preprocessing approaches, dental CBCT images were analyzed to calculate BV/TV; the BV/TV value decreased from 60.27 ± 9.81% (Group 1) to 44.10 ± 12.55% (Group 4). The BV/TV measuring result were significantly different from those obtained through micro-CT. Micro-CT image analysis indicated that the TbTh was 0.20 ± 0.02 mm (group 0); by including image preprocessing approaches into dental CBCT image, we determined that the TbTh decreased from 1.15 ± 0.37 mm to 0.65 ± 0.08 mm (group 1 to 4). Micro-CT image analysis indicated that the TbN was 1.18 ± 0.37 mm (group 0); by including image preprocessing approaches into dental CBCT image, the TbN increased from 0.55 ± 0.09 mm to 0.74 ± 0.12 mm (group 1 to 3) and subsequently decreased to 0.67 ± 0.15 mm (group 4). Some trabecular bone images might be removed during the removal of suppositional air pixels. However, no significant difference was observed between Group 3 and Group 4. Micro-CT image analysis indicated that TbSp was 0.72 ± 0.22 mm (group 0); by including image preprocessing approaches into dental CBCT image, the TbSp decreased from 0.94 ± 0.14 mm to 0.75 ± 0.16 mm (group 1 to 3) and subsequently increased to 0.85 ± 0.22 mm (group 4). Similarly, no significant difference was observed between Group 3 and Group 4. In other words, TbN and TbSp changed after the sharpening and despeckable noise procedure and local thresholding procedure. However, the suppositional air images removal procedure did not influence TbN and TbSp. The estimates of the four trabecular bone microarchitectural parameters obtained using dental CBCT (Group 4) were significantly different from those obtained using micro-CT (Group 0).

**Table 1:** Measurements of trabecular bone microarchitectural parameters based on the micro-CT and dental CBCT images

Scanning machine	Group	Microarchitectural parameters of trabecular bone							
		BV/TV (%)		TbTh (mm)		TbN (1/mm)		TbSp (mm)	
		Mean ± SD	Range	Mean ± SD	Range	Mean ± SD	Range	Mean ± SD	Range
Micro-CT	0	23.85 ± 7.83	9.04–40.18	0.20 ± 0.02	0.16–0.26	1.18 ± 0.37	0.45–1.99	0.72 ± 0.22	0.41–1.32
Dental CBCT	1	60.27 ± 9.81	36.27–87.28	1.15 ± 0.37	0.77–2.72	0.55 ± 0.09	0.32–0.69	0.94 ± 0.14	0.79–1.41
	2	60.58 ± 9.13	32.42–79.16	0.88 ± 0.20	0.62–1.59	0.71 ± 0.12	0.45–0.95	0.77 ± 0.17	0.57–1.34
	3	58.87 ± 6.54	36.70–67.67	0.81 ± 0.11	0.61–1.23	0.74 ± 0.12	0.44–0.98	0.75 ± 0.16	0.56–1.23
	4	44.10 ± 12.55	16.90–60.93	0.65 ± 0.08	0.50–0.84	0.64 ± 0.15	0.33–0.98	0.85 ± 0.22	0.56–1.38

### Relationship between the trabecular bone microarchitectural parameters, micro-CT, and dental CBCT

Table 2 presents the relationship between the estimates of the four trabecular bone microarchitectural parameters obtained through micro-CT and those obtained through dental CBCT. The BV/TV for Group 1 and Group 2 obtained through CBCT were not correlated with that for Group 0 obtained through micro-CT (*p* > 0.05); however, the BV/TV for Group 3 was moderately and positively correlated with Group 0 (*r* = 0.49, *p* = 0.006) and Group 4 was highly positively correlated with Group 0 (*r* = 0.87, *p* < 0.001). The TbTh for Groups 1, 2, and 4 were not correlated with Group 0 (*p* > 0.05); although the TbTh for Group 3 was slightly and negatively correlated with that for Group 0 (*r* = − 0.36, *p* = 0.047), the *p* value was close to 0.05. The TbN for Group 1 was not correlated with that for Group 0 (*p* > 0.05); however, the TbN for Group 2 was moderately and positively correlated with that for Group 0 (*r* = 0.66). The TbN for Group 3 and 4 were significantly and positively correlated with those for Group 0 (Group 3 and Group 0, *r* = 0.75; Group 4 and Group 0, *r* = 0.80). When more image preprocessing steps were performed, the correlation was stronger. The TbSp for Group 1 to 4 were significantly and positively correlated with Group 0 (the *r* values were between 0.78 and 0.93).

**Table 2:** Correlation between the estimates for four trabecular bone microarchitectural parameters obtained through micro-CT and those obtained through CBCT

Comparison		Trabecular bone microarchitecture			
		BV/TV	TbTh	TbN	TbSp
Group 0 vs Group 1	*r*	−0.15	−0.14	0.29	0.78
	P^a^	0.412	0.455	0.117	< 0.001*
Group 0 vs Group 2	*r*	0.16	−0.33	0.66	0.89
	P^a^	0.386	0.069	< 0.001*	< 0.001*
Group 0 vs Group 3	*r*	0.49	−0.36	0.75	0.93
	P^a^	0.006*	0.047*	< 0.001*	< 0.001*
Group 0 vs Group 4	*r*	0.87	0.29	0.80	0.88
	P^a^	< 0.001*	0.116	< 0.001*	< 0.001*

^a^ Paired T-tests

*Statistical significance (*P* < 0.05)

## Discussion

The survival rates of dental implants were considerably influenced by bone quality and quantity at the dental implant sites [25–28]. Understanding the trabecular bone microarchitecture of the jawbone at a dental implant site prior to dental implant surgery is useful for choosing a surgery method or dental implant. In recent years, numerous researchers have used dental CBCT as a method to assess jawbone density prior to dental implant surgery. However, the use of dental CBCT for measuring trabecular bone microarchitectural parameters was limited due to not-enough resolution, which may cause by the large focal spot size, limited number of projections, and partial volume effect of the dental CBCT. Other studies have only indicated that estimates of trabecular bone microarchitectural parameters obtained using dental CBCT were correlated with those obtained using micro-CT; however, large differences were observed between estimates obtained through dental CBCT and those obtained through micro-CT. Few studies have explored the binary image method by using dental CBCT to analyze the trabecular bone microarchitecture; the present study was one of them. This study proposed a sound image preprocessing approach to enhance the capability of dental CBCT to predict trabecular bone microarchitectural parameters.

In 2009, Bouxsein et al. [7] indicated that BV/TV, TbTh, TbN, and TbSp were crucial indicators for assessing trabecular bone microarchitectural parameters. To analyze the trabecular bone microarchitecture, commercially available software packages CTAn (SkyScan, Aartselaar, Belgium) and Scanco (Scanco, Brüttisellen, Switzerland) may be used; in addition, the open-source iamgeJ/BoneJ is widely used [2, 29, 30]. In 2010, Doube et al. [8] determined that the three software packages yielded similar results. In the present study, to avoid measurement errors yielded by various software packages used to obtain estimates of trabecular bone microarchitectural parameters through micro-CT and dental CBCT, we first adopted the ImageJ software package to align micro-CT and dental CBCT and subsequently employed CTAn (SkyScan, Aartselaar, Belgium) to measure trabecular bone microarchitectural parameters in micro-CT and dental CBCT images.

In previous studies on the use of dental CBCT to measure bones, researchers have employed the human cadaveric jawbones [14, 22, 23], human dry jawbones [29–31], or artificial bones [2]. Fresh human cadaveric jawbone cannot be easily obtained. Therefore, in this study, we used fresh bovine vertebrae cancellous bone specimens that can be easily obtained as research samples. Bovine vertebrae bone specimens are large; thus, cortical bones can be easily removed and cancellous bones preserved. Naitoh et al. [31] placed a dry mandible in water for dental CBCT, simulating the surrounding soft tissues. Kang et al. [32] wrapped a cadaveric jawbone in wet tissue for dental CBCT. In the present study, we used bovine vertebrae cancellous bone specimens as research samples; the bone specimens were wrapped in wet gauze and medical tape and were placed at − 20 °C in a constant-temperature refrigerator. Prior to imaging, the bone specimens were fully defrosted at room temperature. Therefore, a small area outside each bone specimen contained water and was used to mimic soft tissues.

In this study, we chose fresh bovine vertebrae cancellous bone specimens. Through micro-CT, the estimates of trabecular bone microarchitectural parameters obtained using the bovine vertebrae cancellous bone specimens were slightly different from those obtained using human jawbone specimens in previous studies. In the present study where micro-CT was used to measure bone specimens, the BV/TV (23.85 ± 7.83%) was smaller than that obtained through bone biopsy on a patient’s jawbone prior to dental implant surgery (48.7 ± 17.5% (18) and 31.42 ± 10.12% (19)). In previous studies where human cadaveric jawbone specimens were used, the BV/TV was between 18.53 ± 8.17% and 34.39 ± 5.41%. Our results were within this range. In addition, the TbTh (0.20 ± 0.02 mm), TbN (1.18 ± 0.37 1/mm), and TbSp (0.72 + 0.22 mm) measured in the present study were similar to those values (0.16 + 0.41 mm – 0.28 + 0.10 mm for TbTh, 0.77 + 0.27 1/mm – 2.2 + 0.72 1/mm for TbN, and 0.3 + 0.1 mm – 0.83 + 0.17 mm for TbSp) obtained using a patient’s jawbone or human cadaveric jawbone. The use of bovine vertebrae cancellous bone specimens produced representative results.

Micro-CT has been considered the gold standard for assessing trabecular bone microarchitecture [5, 7]. However, the binary image method can influence the measurement results of micro-CT. Currently, a widely used binary image method is the grayscale threshold approach. In this approach, a grayscale threshold value is set, and pixels with grayscale values greater than the threshold value are considered hard tissues and those with grayscale values lower than the threshold value are considered soft tissues. This method is simple and can be applied in general situations. The threshold value for binarization can influence measurement outcomes [19, 20]; however, because the resolution of micro-CT is higher than that of dental CBCT, the partial volume effect produced by micro-CT is smaller than that produced by dental CBCT. Therefore, the influence of threshold value variation during the micro-CT binary image procedure can be ignored.

Previously, researchers have proposed various binary image methods for dental CBCT. Naitoh et al. [31] used five different grayscale threshold values between cortical bone and water values to binarize images. Ibrahim et al. [22, 33–35] used the automated histogram analysis in CTAn software to analyze trabecular bone microarchitectural parameters. Haung et al. [36] adopted the adaptive thresholding algorithm to determine the binary threshold value. Klintstrom et al. [37] employed the automated three-dimensional (3D) region growing algorithm to segment bone from other tissues. Panmekiate et al. [29] used the moment method proposed by Tsai et al. [38] to determine the threshold value. In the present study, four preprocessing methods were used during dental CBCT image binarization, namely filter sharpening, despeckable noises removal, local thresholding, and suppositional air images removal. These methods can be used to effectively segment trabecular bone tissues from whole specimen images. The experiment results indicated that through the use of image preprocessing approaches, dental CBCT can accurately measure trabecular bone microarchitectural parameters.

In this study, the correlation coefficient between the BV/TV obtained through dental CBCT and that obtained through micro-CT was 0.49 (i.e., the correlation between Group 0 and Group 3). The value was lower than those found in previous studies (Table 3.). Using human cadaveric jawbone specimens, the correlation coefficients obtained by Parsa et al. [14], Van Dessel et al. [24], and Kim et al. [23] were 0.82, 0.76–0.89, and 0.61, respectively. For Group 4, the correlation coefficient between the BV/TV obtained through dental CBCT and that obtained through micro-CT was 0.87, which was greater than the values found in previous studies. Considering TbN and TbSp, the correlation coefficients between Group 4 and Group 0 were also greater than those found in previous studies. Considering TbTh, Group 4 was not correlated with Group 0 (*p* > 0.05). The main reason was that sharpening images and removing despeckable noises resulted in losing thin trabecular bones. In addition, previous studies have determined that the TbTh obtained through dental CBCT was not correlated with that obtained through micro-CT [23, 24].

**Table 3:** Pearson relation coefficient (*r*) between micro-CT and dental CBCT measurements in this study and previous studies

References	Correlation between micro-CT and dental CBCT measurement				Sample source (sample number)
	BV/TV	TbTh	TbN	TbSp	
This study	0.87*	0.29	0.80*	0.88*	Fresh bovine vertebrae cancellous bone (30)
Ibrahim et al. [22]	NA	0.82*	0.85*	0.94*	Human cadaveric mandible (24)
Parsa et al. [14]	0.82*	NA	NA	NA	Human cadaveric mandible (20)
Van Dessel et al. [24]	0.76–0.89*	0.21–0.57	0.72–0.86*	0.61–0.84*	Human cadaveric mandible (8)
Kim et al. [23]	0.61*	0.05	0.25*	0.58*	Human cadaveric jawbone (68)
Panmekiate et al. [29]	0.80	0.52	NA	0.55	Human cadaveric mandible (20)

*P* value * < 0.05

*NA* Not available

The limitations of this study must be considered when drawing conclusions. First, because fresh human cadaveric jawbone cannot be easily obtained, we could only use fresh bovine vertebrae cancellous bone specimens to mimic cadaveric jawbone. Second, in this study, an in vitro scan condition was applied to bone specimens, which could be clearer than an in vivo scan condition because no interference from other bones and soft tissues affected the imaging. Third, we used only a single dental CBCT machine and scan protocol (85 kV, 5 mA, and a voxel resolution of 100 μm). In the future, other brands of dental CBCT machines with various resolutions and scan settings should be employed to fully explore this topic.

## Conclusion

The estimates of trabecular bone microarchitectural parameters obtained through dental CBCT differed significantly from those obtained through micro-CT. The image preprocessing approaches of sharpening images, removing despeckable noises, applying local binary threshold, and removing air pixels were used to optimize image binarization. Therefore, the difference between the trabecular bone microarchitectural parameters obtained using dental CBCT and those obtained using micro-CT were reduced and the efficacy of using dental CBCT to predict BV/TV, TbN, and TbSp was increased.

## Abbreviations

BV/TV: Bone volume fraction
CBCT: Cone-beam computed tomography
HU: Hounsfield units
micro-CT: Micro-computed tomography
ROI: Regions of interest
TbN: Trabecular number
TbSp: Trabecular separation
TbTh: Trabecular thickness
